# Aspirin and atenolol enhance metformin activity against breast cancer by targeting both neoplastic and microenvironment cells

**DOI:** 10.1038/srep18673

**Published:** 2016-01-05

**Authors:** Giovanna Talarico, Stefania Orecchioni, Katiuscia Dallaglio, Francesca Reggiani, Patrizia Mancuso, Angelica Calleri, Giuliana Gregato, Valentina Labanca, Teresa Rossi, Douglas M. Noonan, Adriana Albini, Francesco Bertolini

**Affiliations:** 1 Laboratory of Hematology-Oncology, European Institute of Oncology, Milan, Italy; 2 Research and Statistics Department, IRCCS “Tecnologie Avanzate e Modelli Assistenziali in Oncologia” Arcispedale S. Maria Nuova, Reggio Emilia, Italy; 3 Scientific and Technology Pole, IRCCS MultiMedica, Milan, Italy; 4 Department of Biotechnology and Life Sciences, University of Insubria, Varese, Italy

## Abstract

Metformin can induce breast cancer (BC) cell apoptosis and reduce BC local and metastatic growth in preclinical models. Since Metformin is frequently used along with Aspirin or beta-blockers, we investigated the effect of Metformin, Aspirin and the beta-blocker Atenolol in several BC models. *In vitro*, Aspirin synergized with Metformin in inducing apoptosis of triple negative and endocrine-sensitive BC cells, and in activating AMPK in BC and in white adipose tissue (WAT) progenitors known to cooperate to BC progression. Both Aspirin and Atenolol added to the inhibitory effect of Metformin against complex I of the respiratory chain. In both immune-deficient and immune-competent preclinical models, Atenolol increased Metformin activity against angiogenesis, local and metastatic growth of HER2+ and triple negative BC. Aspirin increased the activity of Metformin only in immune-competent HER2+ BC models. Both Aspirin and Atenolol, when added to Metformin, significantly reduced the endothelial cell component of tumor vessels, whereas pericytes were reduced by the addition of Atenolol but not by the addition of Aspirin. Our data indicate that the addition of Aspirin or of Atenolol to Metformin might be beneficial for BC control, and that this activity is likely due to effects on both BC and microenvironment cells.

A variety of preclinical and epidemiological studies have recently indicated that the biguanide Metformin (Met), currently used worldwide by type 2 diabetic patients, can reduce the incidence and severity of several types of cancer^1^,^2^,^3^,^4^,^5^,^6^,^7^,^8^,^9^,^10^,^11^,^12^,^13^,^14^,^15^,^16^. Interestingly, cancers rising in tissues and/or organs rich in white adipose tissue (WAT) such as the digestive-tract, the breast and the prostate, seem to receive most benefit from Met administration^1^,^2^,^3^. Along this line, we have recently reported that Met can target *in vitro* and *in vivo* both breast cancer (BC) and WAT progenitor cells^17^.

Aspirin (Asp) and beta blockers are currently used together with Met by millions of patients with diabetes and cardiovascular diseases (CVD)^12^,^13^,^15^. However, to date there are no reports on the effect of the combination of Met plus Asp and of Met plus beta blockers *in vitro* and in preclinical BC models. Here we report about the combinatory effect of these three drugs over BC cells (triple negative and endocrine-responsive) and WAT progenitor cells *in vitro* and *in vivo*.

The AMPK/mTOR pathway and the complex I of the respiratory chain are currently considered the most relevant possible targets of Met activity in BC, and both were investigated in our *in vitro* studies^18^,^19^. Furthermore, we observed the *in vivo* effects of Met, Asp and the beta blocker Atenolol (At) in orthotopic local and metastatic models of triple negative and HER2+ BC. Considering the known effect of Met and of Asp over immune cells^20^,^21^, we also investigated *in vivo* both immune-competent and immune-deficient BC models.

Our studies on Met activity towards BC and WAT progenitors, found that another biguanide, Phenformin (Phe), was significantly more active than Met *in vitro* and *in vivo*^17^. However, Phe has several clinical side effects that caused its withdrawn from clinical use in diabetes^12^,^13^. In the present report, data obtained with Phe are used as a reference of how the addition of Asp or At can increase the effect of Met *in vitro* and *in vivo*.

## Results

### *In vitro*, Asp synergizes with Met in inducing apoptosis of triple negative and endocrine-sensitive BC cells

**Figure 1:**
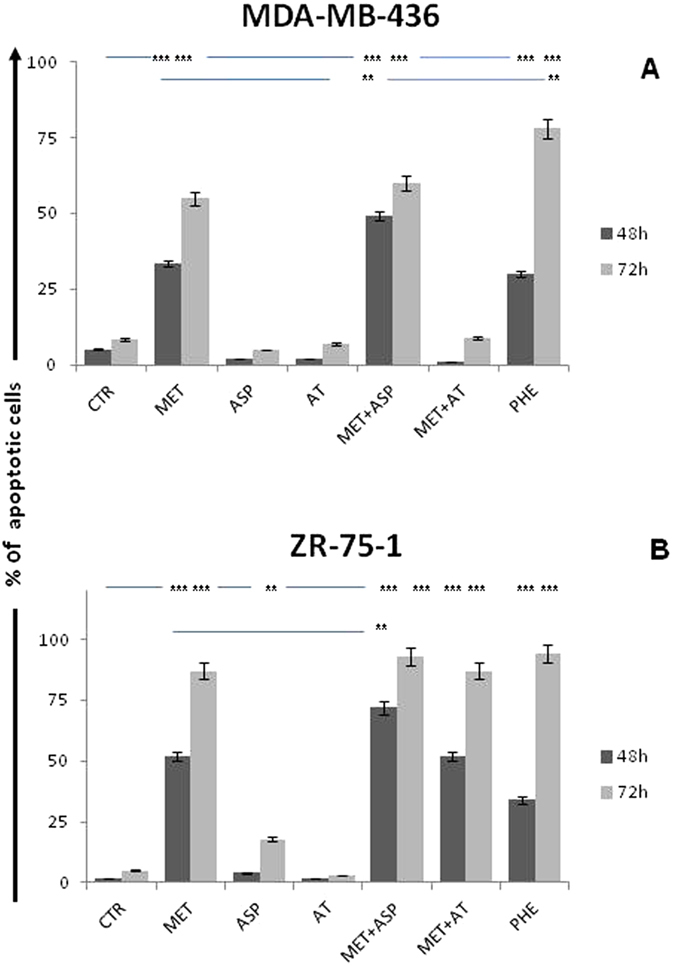
Induction of BC cells apoptosis *in vitro* in culture with Met, Asp, At and Phe. Panels (**A,B**) show the effect of Met, Asp and At on MDA-MB-436 and ZR-75-1 cells, respectively. Cell apoptosis was analyzed by flow cytometry, and shown as percent viable cells. The addition of Asp to Met significantly increased the frequency of apoptotic BCs. *P < 0.05, **P < 0.01, ***P > 0.005 vs controls or Met alone.

To evaluate the inhibitory effect of Met along with Asp or At on human BC cells *in vitro*, the BC cell lines MDA-MB-436 and ZR-75-1 were treated with different drug combinations including 10 mM Asp, 500 uM At, 10 mM Met, and 2 mM Phe for 48 and 72 hours. Results of preliminary studies investigating the *in vitro* activity of different concentrations of Met and Asp are reported in Supplementary Fig. 1 A and B. As expected^22^, flow cytometry indicated that in both BC cell lines, Met increased the percentage of apoptotic cells (Fig. 1). Alone, Asp and At did not or marginally increased the frequency of apoptotic BC cells when compared to controls. The addition of Asp to Met significantly increased the frequency of apoptotic MBA-MB-436 triple negative BC cells after 48 h of culture when compared to results observed using Met alone (p < 0.01). Similarly, the addition of Asp to Met significantly increased the frequency of apoptotic endocrine-sensitive ZR-75-1 BC cells after 48 h of culture when compared to Met alone (p < 0.01). When compared to the percentage of BC apoptotic cells induced in culture by Phe, a biguanide known to be more toxic but also more active than Met^17^, the combination of Met and Asp showed significantly more apoptosis after 48 h culture. The addition of At to Met, on the other hand, did not show an additive effect *in vitro* under these culture conditions. Cell proliferation data are reported in Supplementary Fig. 2A,B.

### Asp cooperates with Met to activate AMPK in BC cells

**Figure 2:**
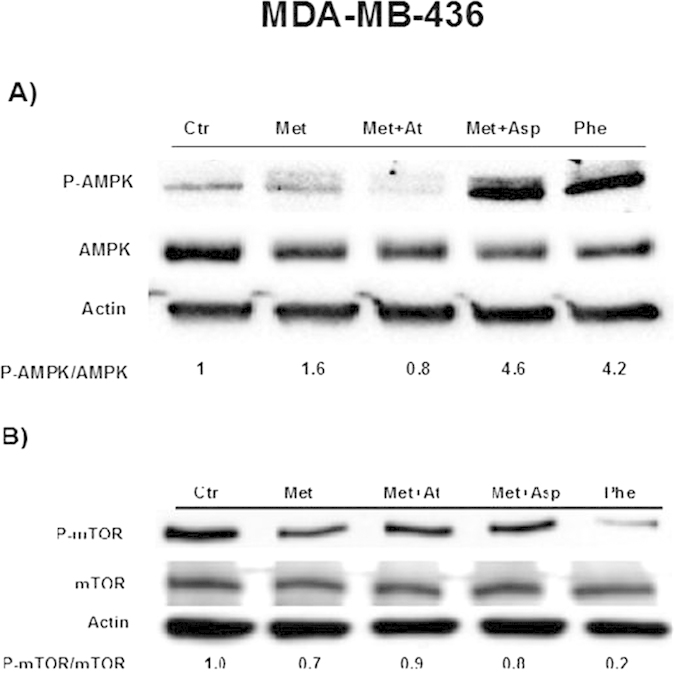
(Panel A) AMPK activation by Met or Phe alone or by Met in combination with Asp or At in triple negative MDA-MB-436 BC cells. (**Panel B**) mTOR activation in MDA-MB-436 cells treated with the same stimuli as in A. For each blot, the levels of p-AMPK over total AMPK and p-mTOR over total mTOR were quantified by ImageJ and are represented by numbers below the bands. The experiments were performed in triplicate.

**Figure 3:**
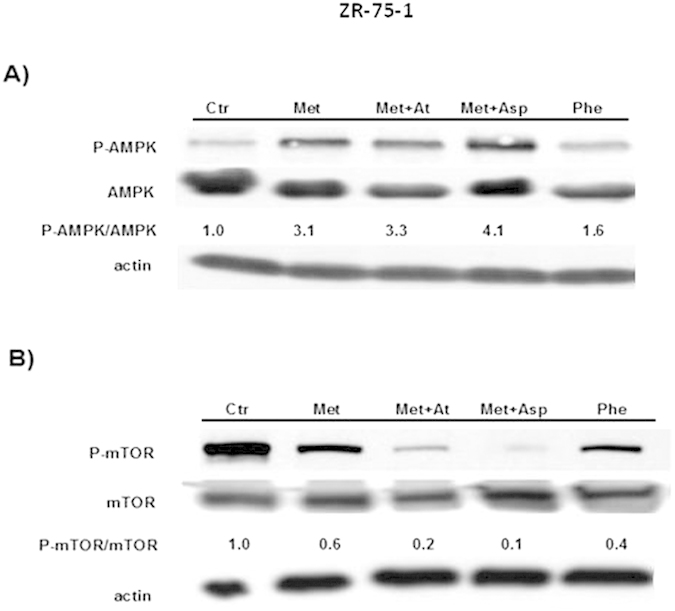
(Panel A) AMPK activation by Met or Phe alone or by Met in combination with Asp or At in endocrine-sensitive ZR-75.1 BC cells. (**Panel B**) mTOR activation in ZR-75.1 cells treated with the same stimuli as in A. For each blot, the levels of p-AMPK over total AMPK and p-mTOR over total mTOR were quantified by ImageJ and are represented by numbers below the bands. The experiments were performed in triplicate.

We observed an increased level of p-AMPK over total AMPK after 24 h culture in both MDA-MB-436 (Fig. 2A) and ZR-75-1 (Fig. 3A) BC cells treated with Met+Asp compared to controls and to cells treated with Met alone. We also observed a different effect of Met and Phe on these cells. Phe activates AMPK in MDA-MB-436 triple negative BC cells more efficiently than in endocrine-sensitive ZR-75-1 BC cells. We have recently shown that AMPK activation by Met in BC cells results in the inhibition of the mTOR signaling pathway, which in turn controls protein homeostasis and cell survival^17^. In order to confirm this, we analyzed p-mTOR and total mTOR levels in the same cells (Figs 2B and 3B). Overall, p-mTOR levels are reduced in Met or Phe-treated cells (in all the different combinations, in both MDA-MB-436 and ZR-75.1 cells), indicating that Met or Phe-activated AMPK may directly inhibit mTOR activation. The only exception was observed in MDA-MB-436 cells, where the addition of At to Met-treated cells restored p-mTOR levels similar to those of control, indicating that Met+At mediated activation of AMPK may not lead to mTOR inhibition at this time point (Fig. 2B). This opens up the possibility that At exerts its effects by activating/inhibiting other mediators in these cells.

### Asp and At collaborate with Met to activate AMPK in WAT progenitor cells

**Figure 4:**
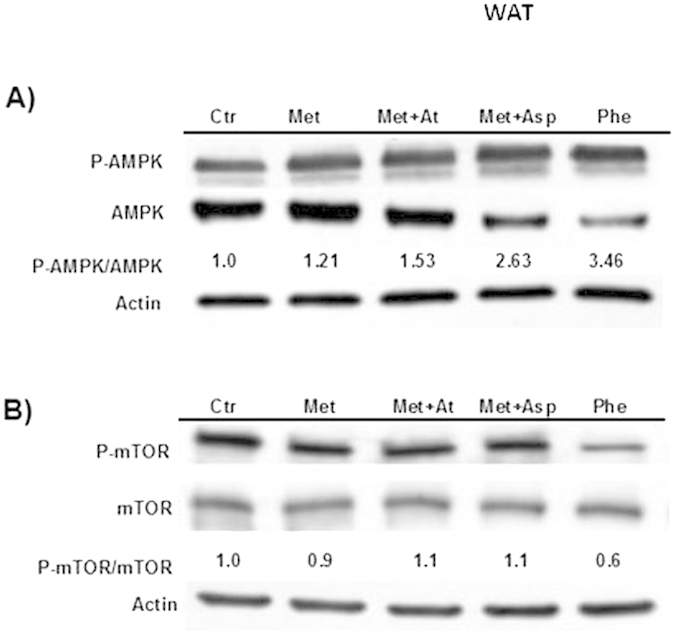
(Panel A) AMPK activation by Met or Phe alone or by Met in combination with Asp or At in WAT progenitors. (**Panel B**) mTOR activation in WAT progenitors treated with 20 mM Met and 0.5 mM Phe for 16 h as in A. For each blot, the levels of p-AMPK over total AMPK and p-mTOR over total mTOR were quantified by ImageJ and are represented by numbers below the bands. The experiments were performed in triplicate.

We treated WAT progenitor cells with 20 mM Met and 0.5 mM Phe for 16 h and analyzed both AMPK and mTOR activation. The combination of Met with At or Asp further activates AMPK as compared to Met alone, and Met+Asp treatment showed the highest phosphorylation status of the protein (Fig. 4A). In order to analyze the downstream molecular effects of AMPK activation in WAT progenitors following treatment with Met or Phe alone or with Met in combination with Asp or At, we performed a western blotting to measure phospho-mTOR levels over total mTOR in treated WAT progenitor cells as compared to control. While treatment with Met (alone or in combination with Asp or At) did not reduce p-mTOR levels, we observed a reduced activation of mTOR over total mTOR in WAT progenitors treated with Phe alone at 16 h (Fig. 4B).

We also analyzed the activation of the downstream mediators of mTOR pathway, 4E-BP1 and p70S6K. As shown in Supplementary Fig. 3, we observed the inhibition of both 4E-BP1 and p70S6K phosphorylation in ZR-75.1 cells only. Since we did not observe the same effect in MDA-MB-436 cells, AMPK activation might or might not be followed by inhibition of mTOR pathway in BC cells. This is possibly due to pathways different from that of mTOR, whose analysis has not been included in this manuscript. In addition, AMPK activation is not followed by inhibition of mTOR pathway in WAT progenitors.

### Met in combination with Asp and At inhibits complex I of the respiratory chain

**Figure 5:**
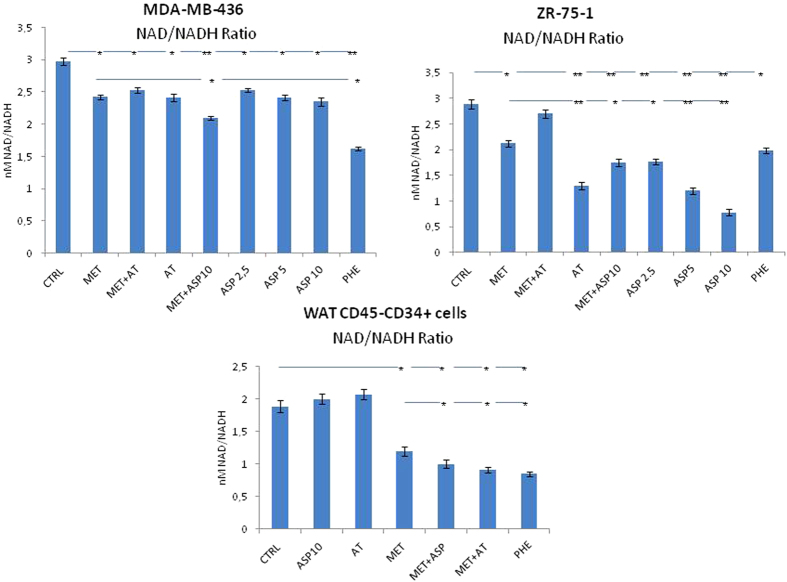
As NAD+/NADH is a known target of biguanide activity, NAD(H) was analyzed in CD34+ WAT-derived cell and in MDA-MB-436 and ZR-75-1 BC cells using a Fluorescent NAD/NADH Detection Kit (n = 7). Asp and At alone did not show any inhibition of complex I of the respiratory chain in MDA-MB-436 and CD45-CD34+ WAT-derived progenitor cells. On the other hand, in ZR-75-1 cell lines Asp inhibits complex I in a dose dependent manner, and similar effect is obtained using At alone. *P < 0.05, **P < 0.01, ***P > 0.005 vs controls or Met alone.

One of the primary cellular targets of Met and Phe is the mitochondrion^23^, where they directly inhibit complex I NADH dehydrogenase of the respiratory chain possibly causing a decrease in cAMP production and the activation of AMPK. As NAD+/NADH is another possible target of biguanide activity, NAD(H) was analyzed both in CD45-CD34+ WAT-derived progenitor cells and in BC cells. As shown in Fig. 5 (and in Supplementary Fig. 4), the addition of Asp to Met increased the inhibition of complex I of the respiratory chain in MDA-MB-436 triple negative BC cells and in ZR-75-1 endocrine BC cells, whereas the addition of At to Met had no effect. On the other hand, in WAT progenitors, the addition of Asp or of At to Met similarly increased the inhibition of complex I.

### At increased the activity of Met against the local and metastatic growth of HER2+ and triple negative BC. Asp increased the activity of Met only in immune-competent models

**Figure 6:**
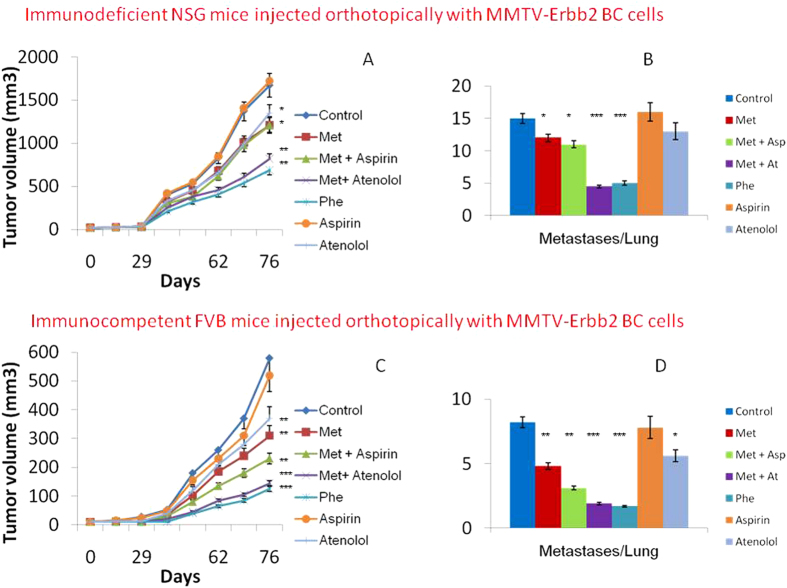
Tumor volumes (panel A) and the number of metastases per lung after mastectomy (panel B) in immunodeficient NSG mice injected orthotopically with murine MMTV-Erbb2 BC cells and treated with Met, Phe, Asp, At, Met and Asp, Met and At vs controls. Tumor volumes (panel C) and the number of metastases per lung after mastectomy (panel D) in immunocompetent FVB mice injected orthotopically with murine MMTV-Erbb2 BC cells and treated with Met, Phe, Asp, At, Met and Asp, Met and At vs controls. *P < 0.05, **P < 0.01, ***P > 0.005 vs controls.

**Figure 7:**
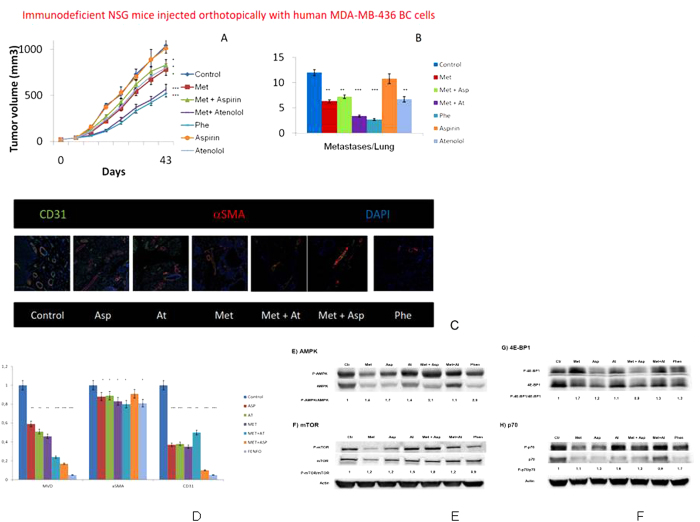
Tumor volumes (panel A) and the number of metastases per lung after mastectomy (panel B) in immunodeficient NSG mice injected orthotopically with human MDA-MB-436 triple negative BC cells and treated with Met, Phe, Asp, At, Met and Asp, Met and At vs controls. Representative vessels in tumors of immunocompetent FVB mice injected with murine MMTV-Erbb2 BC cells (panel C). Using immunofluorescence and confocal microscopy, z-stack images depicted CD31+ endothelial cells (green), alpha-SMA+ pericytes (red) and DNA (DAPI, blue). Squares are representative of an area of 775 × 775 μm3. Panel D shows the relative frequency of microvessel density (MVD), alphaSMA+ and CD31+ cells in the tumors of immunocompetent FVB mice injected with murine MMTV-Erbb2 BC cells (panel C). Results observed in immunodeficient NSG mice injected with murine MMTV-Erbb2 BC cells or human MDA-MB- 436 BC cells were not significantly different (data not shown). Panel E shows AMPK activation in MDA-MB-436-derived tumors generated in immunodeficient NSG mice injected with human MDA-MB-436 BC cells and treated with Met or Phen alone or in combination with Aspirin (Asp) and/or Atenolol (At). Panel F shows mTOR activation in the same tumors analyzed in A. Panel G shows Activation of 4E-BP1 in the same tumors analyzed in A. Panel H shows p70 activation in the same tumors analyzed in A. For each blot, the levels of phosphorylated protein over total protein were quantified by image-j, compared to those of control and represented by numbers below the bands. Actin was used as loading control. *P < 0.05, **P < 0.01, ***P > 0.005 vs controls.

We studied the effect of Met plus Asp and of Met plus At in different BC models. As shown in Fig. 6A,B and in Fig. 7A,B, in immune-deficient (NGS) mice, Asp was not additive *in vivo* to the activity of Met against local and metastatic BC growth in both HER2+ and triple negative models. In the same immune-deficient NGS mice, the addition of At significantly increased the activity of Met against local and metastatic HER2+ (Fig. 6) and triple negative (Fig. 7) local and metastatic BC growth.

As shown in Fig. 6C,D, in immune-competent (FVB) mice orthotopically injected with HER2+ BC cells, the addition of both At and Asp increased the activity of Met against local and metastatic BC growth. When used alone, the activity of Asp and of At were significantly lower than those of Met plus Asp and of Met plus At.

### Met, Asp and At targeted the endothelial compartment of BC vessels and generated dysplastic vessels

As shown in Fig. 7C,D, treatment with Met resulted in a significant reduction in tumor MVD, and the addition of Asp and At to Met further decreased MVD. By means of polychromatic immunofluorescence we found that Met+Asp and of Met+At significantly reduced the endothelial (CD31+) cell component of tumor vessels, whereas αSMA+ pericytes were reduced by Met+At but not by Met+Asp. As already reported^17^, after Met administration tumor vessels displayed a branching and dysplastic phenotype (Fig. 7C), and the effect of Phe was quantitatively increased when compared to Met.

No significant differences in the activity of Met and Phe were observed in immune-deficient compared to immune-competent models or in triple negative vs HER2+ BC models (data not shown).

## Discussion

Met, Asp and beta blockers are currently used worldwide by millions of patients with diabetes and cardiovascular diseases (CVD). In the past few years, the use of Met has been associated in preclinical models and in clinical retrospective studies in diabetic patients with a reduced incidence and severity of several types of cancer, including BC^1^,^2^,^3^,^4^,^5^,^6^,^7^,^8^,^9^,^10^,^11^,^12^,^13^,^14^,^15^,^16^. Although Met doses used in the present *in vivo* preclinical studies are higher than those used in human diabetic patients, the current dosage is in the range that was previously observed to prolong lifespan in mice^24^. Further studies are needed to define the best dosage for Met in clinical use for oncology.

Three large observational studies have shown a survival benefit in BC patients using Asp or other non steroidal anti inflammatory drugs (NSAIDs,^25^,^26^,^27^). Similarly, some recent retrospective clinical studies have suggested that the use of beta blockers might be beneficial for some BC patients^28^,^29^,^30^,^31^. In spite of these data and of the frequent combinatory clinical use of Met and Asp or of Met and beta blockers in patients affected by diabetes and CVD, very few data are available about cancer incidence and severity in preclinical models and in patients treated with these combinatory treatments. In preclinical models, pancreatic cancer prevention seems to be possible by combining Met and Asp^32^. Din *et al.*^33^ have shown that Met and Asp can control colorectal cancer cell growth by targeting AMPK and mTOR signaling pathways, as well as inflammation.

The choice to study together Met and Asp was also due to the increasing evidence that molecular targets of Met and Asp are multiple and not only restricted to AMPK. Also, cancer and microenvironment cells are differently targeted (in multiple pathways) by Met and Asp.

We have recently reported that Met inhibits BC cells *in vitro* and reduces angiogenesis, local and metastatic BC progression *in vivo* by targeting both cancer and microenvironment WAT cells^17^,^22^. Considering that Met is frequently administered along with Asp and beta-blockers in diabetic, CVD and obese patients, we tested *in vitro* and in preclinical local and metastatic BC models the association of Met with Asp and of Met with a beta blocker. To minimize the use of animals, in preclinical studies we focused on models of the most aggressive types of breast neoplasia, namely triple negative and HER2+ diseases. At was chosen as a beta blocker for its safety profile in long-term administration along with Met. To the best of our knowledge, this is the first report investigating these drug combinations in BC cells *in vitro* and in orthotopic local and metastatic BC models of triple negative and HER2+ disease (*in vitro* data with HER2+ cells are reported in Supplementary Fig.5).

In our study on Met activity against BC *in vitro* and *in vivo*, we also reported that another biguanide, Phe (withdrawn from clinical use for toxicity, mainly due to acidosis) was significantly more active than Met, both *in vitro* and *in vivo*. However, the safety profile of Phe renders its possible future development as an anticancer drug unclear. Data obtained with Phe are reported here as a reference of how the addition of Asp or At can increase the effect of Met *in vitro* and *in vivo*.

AMPK, which is activated by Met and by Asp in neoplastic cells^34^,^35^, was synergistically activated when these drugs were used in combination. This synergy was observed both in BC cells and in purified WAT progenitors. It is known that mTOR signaling is dependent on AMPK activation status, being primarily blocked when AMPK is activated. However, in our hands mTOR inhibition failed to occur in an AMPK-dependent manner. We did not observe inhibition of mTOR phosphorylation in WAT progenitors treated with Met+Asp or Met+At. On the other hand, it was strongly reduced in Met+Asp treated BC cells. Taken together, our results suggest that Met and Asp synergistically activate AMPK in both BC cells and WAT progenitors, whereas the mTOR pathway seems to be inhibited by combination treatment only in BC cells.

As recent data underlines that also the mitochondrion might be among of the primary cellular targets of Met and Phe^18^,^19^, we investigated NAD(H) *in vitro* in BC and in WAT-derived progenitor cells. Adding Asp to Met increased the inhibition of complex I of the respiratory chain both in triple negative and in endocrine-responsive BC cells. On the other hand, the addition of At to Met had no effect. At variance, *in vitro* studies on WAT progenitor cells, the addition of At to Met increased the inhibition of complex I at levels similar to those observed when Asp was added to Met.

We then investigated whether Asp or At might add to the activity of Met against angiogenesis, local and metastatic BC growth in triple negative and in HER2+ preclinical models. Despite the *in vitro* results showing an additive effect of Asp to Met, in immune-deficient mice Asp did not further increase to the activity of Met against angiogenesis, local and metastatic BC growth in both HER2+ and triple negative models. In contrast, in the same immune-deficient models At significantly increased the activity of Met against local and metastatic BC, both HER2+ and triple negative preclinical models. In immune-competent models, the addition of Asp increased the activity of Met against local and metastatic HER2+ BC growth, as did the addition of At. Notably, the addition of At to Met led to an inhibition of local and metastatic BC growth similar to the data observed after the administration of the more toxic biguanide, Phe.

The addition of At to Met appeared to be somewhat protective in some *in vitro* studies (Figs 1A and 5A,B). On the other hand, this combination was more effective *in vivo* in reducing tumor growth and metastases (Figs 6A,B and 7A,B). These data raise the possibility that this combination has effects on endocrine pathways and/or tumor microenvironment cells/pathways other than those studied and reported in the present manuscript. This observation deserves further studies.

As shown in Fig. 7E, in immune-deficient mice, Asp was additive to Met in activating AMPK. This is in line with what observed *in vitro* (Figs 2 and 3). In addition, as expected, we observed a strong activation of AMPK in Phe-treated mice. As opposite to what observed in local and metastatic BC growth experiments, the combination of At with Met was not additive *in vivo* in terms of activation of AMPK. Interestingly, the fact that p-mTOR levels are either increased or not changed in all the different combinations, indicates that *in vivo*, pathways different from that of mTOR may be involved. This was also confirmed by the levels of p-4E-BP1 and p-p70S6K over total proteins showing the same trend in the different conditions (Fig. 7G,H).

Taken together, our data suggest that some immune cells might play a relevant role to improve the *in vivo* synergy of Asp with Met against BC, whereas the immune system might be less important in the synergy between Met and At. We are trying to generate an adequate immune-competent triple-negative BC model to confirm our findings also for this type of BC. At the same time, we are comparing the efficacy of At with that of other beta-blockers with more favorable safety profiles for long term administration along with Met.

## Methods

### *In vitro* studies

Human WAT was obtained as previously described^36^,^37^ from women undergoing breast reconstruction after the signature of an informed consent. Methods were carried out in accordance with the approved guidelines. The study was approved by the Ethical Committee of the European Institute of Oncology. In brief, samples were centrifuged at 1200 g to remove erythrocytes and leukocytes and subsequently digested in Phosphate Buffered Saline (PBS, Lonza) containing 2 mg/mL of collagenase type I (Sigma Aldrich, St. Louis, MO, USA) and 3.5% bovine serum albumin (BSA; Sigma) at 37 °C with constant shaking for 120 minutes. The digestion was blocked with RPMI 1640 supplemented by 20% FBS (Euroclone, Italy), and a cell pellet was obtained by centrifugation at 1200 g for 10 minutes at 4 °C. The cell suspension was then processed through a 100μm mesh filter to remove undigested tissue and washed twice with incubation buffer (PBS with 2 mM EDTA and 0.5% BSA), always at 4 °C. An aliquot of these cells was labeled for flow cytometry analysis. At least 500.000 total cells per sample were acquired on a flow cytometer equipped with 3 laser (Navios, Beckman Coulter, Pasadena, CA, USA) and analysis was performed by a KALUZA software (Beckman Coulter), after selection of DNA+ (Syto16+) and viable (7-AAD) cells. Analysis gates were set with the aid of “fluorescence minus outcomes” isotype controls.

WAT cell sorting was done as we previously described^17^,^37^. Briefly, CD34+ microbeads-purified cells were labeled with sterile CD45FITC, CD13PE, CD31 PeCy7 and CD34APC monoclonal antibodies, and resuspended in PBS1X/EDTA 2 mM/FBS1% for cell sorting using a three laser Influx high speed cell sorter (BD, Mountain View, CA, USA) equipped with a class I biosafety cabinet.

Samples were continuously cooled to 4 °C and a forward scatter pulse height and side scatter analyses were performed to exclude cell clusters and doublets. A two way cell sorting procedure was performed with a 140 um nozzle with a 5.5 PSI pressure, and with an events rate of 1,000-1,500 events per second, using a sort pure mode. Samples were collected into sterile polypropylene tubes containing 20% FBS, and used for *in vitro* and *in vivo* studies. Analysis was performed using the FACS-Sortware software (BD). WAT EPC and ASC purity was always greater than 96% with a recovery of 70-80%.

Human MDA-MB-436 (triple negative), and ZR-75-1 (ER+ PR+ HER2-), BC cells were purchased from the American Type Culture Collection (Manassas, VA, USA), tested and authenticated by Stem cell Elite ID (Promega) in January 2014 and cultured as previously described^35^,^36^. Murine HER2+ MMTV-Erbb2 BC cells obtained from FVB-Tg (MMTV-Erbb2)NK1Mul/J mice were cultured as previously described^37^.

### Flow cytometry apoptosis assay

Cell lines and sorted WAT progenitors were plated 24 and 16 hours respectively before culture in the presence of vehicle or of different concentrations and combinations of Met, Phe, Asp or At (Sigma) in a 37 °C, 5% CO_2_ humidified incubator (n = 5 per study arm). Cells were collected and analyzed by flow cytometry for apoptosis 48 and 72 hours for BC cells and for 16 hours for WAT after drug supplementation. The nuclear staining Syto16 was used to discriminate between DNA containing cells and cell debris, which were excluded by gating. 7-Amino-actinomycin D (7-AAD) was used to determine the viability status of the cells^17^,^36^,^37^.

### Western blotting

AMPK and phospho-AMPK, as well as phospho-mTOR and mTOR protein expression were examined in BC cells and purified WAT progenitors maintained under different treatment conditions. Briefly, MDA-MB-436 and ZR-75-1 human BC cells and purified human WAT progenitors were treated, respectively, with 10 mM Met or 2 mM Phe for 24 h (BC cells) and 20 mM Met or 0.5 mM Phe for 16 h (WAT) alone or in combination with 10 mM Asp or 500 μM At. Cells were lysed in ripa buffer containing phos-STOP solution (Roche LifeScience). Lysates were then centrifuged at 12,000 rpm at 4 °C for 20 minutes to remove any cell debris. Protein concentrations were determined using Bradford assay (BioRad, BioRad Laboratories Inc., CA, USA). Total tissue lysate were then heated at 90 °C and resolved on a 4–20% gradient Mini-Protean TGX gels (Bio-Rad). Proteins were subjected to PAGE, electrotransferred to nitrocellulose membranes (Amersham Biosciences), incubated in blocking buffer (5% Non-fat dry milk in PBS/0.1% Tween) followed by overnight incubation with the indicated antibodies in PBS-Tween containing 5% (w/v) BSA for 16 h at 4 °C. Antibodies used for western blotting were anti-phospho-AMPK, anti-AMPKa1, anti-phospho-mTOR, anti-mTOR, anti-phospho-4E-BP1, anti-4E-BP1, anti-phospho-p70S6K and anti-p70S6K (all from Cell Signalling, NEB, Hitchen, UK) and anti-actin (Sigma-Aldrich (St. Louis, MO, USA). Detection was performed using anti-mouse or anti-rabbit horseradish peroxidase-conjugated antibody and chemiluminescence with ECL reagent (Amersham Biosciences).

### Studies on complex 1 of the respiratory chain

As NAD+/NADH is another possible target of biguanides activity, NAD(H) was analyzed both in CD45-CD34+ WAT-derived progenitor cells and BC cell lines using the fluorescent NAD/NADH detection kit (Cell Technology, Mountain View, CA, USA) following the manufacturer’s protocol.

### Orthotopic xenografts

Experiments were carried out on immune-deficient NOD SCID IL2RG null (NSG) and immune-competent FVB female mice, 6 to 9-weeks-old. NSG mouse strain were bred and housed under pathogen-free conditions in our animal facilities at the European Institute of Oncology–Italian Foundation for Cancer Research (FIRC) Institute of Molecular Oncology (IEO-IFOM, Milan, Italy). Mice were expanded from breeding pairs kindly donated by Dr. Leonard Shultz, The Jackson Laboratory, Bar Harbor, ME. FVB mice were purchased from Harlan Laboratories (Italy).

Experiments involving animals have been done in accordance with the approved guidelines. In particular, studies were carried out in accordance with the Italian Laws (D.L.vo 116/92 and following additions), which enforce EU 86/609 Directive (Council Directive 86/609/EEC of 24 November 1986 on the approximation of laws, regulations and administrative provisions of the Member States regarding the protection of animals used for experimental and other scientific purposes). Mice have been housed accordingly to the guidelines set out in Commission Recommendation 2007/526/EC - June 18, 2007 on guidelines for the accommodation and care of animals used for experimental and other scientific purposes.

Until March 28, 2014, the Italian legislation did not require a specific ethical review process for all the experiments involving animals. A central (Government) review was required only for particular species (e.g., dogs, cats, and non-human primates) or for experiments done without anesthesia or that will or may cause severe pain. In the other cases, only a notification of the experiments to the Ministry of Health was required. Accordingly, the project was notified to the Ministry of Health.

To produce orthotopic primary human tumors in NSG mice, MDA-MB-436 BC cells were injected in the mammary fat pad as we previously described^36^,^37^. In the second model of orthotopic xenograft transplantation, spontaneous tumors from 36-weeks-old ErBb2-transgenic mice on an FVB background^38^ were dissected and dissociated to generate single cell suspensions in PBS to a final concentration of 10^6^ cells/13 μL. Briefly, after mechanical dissociation with gentle MACS Dissociator (Miltenyi Biotech, Germany), tumors were placed in culture medium (1:1 of Dulbecco’s Modified Eagle’s Medium with high glucose and Ham’s F-12 Nutrient Mixture, EuroClone) containing collagenase (Sigma) and hyaluronidase (Sigma), and digested for 2 h at 37 °C. After lysis of the red blood cells, a single cell suspension was obtained by sequential dissociation of the fragments by gentle pipetting for 3 min in 0.25% trypsin (EuroClone), and then 3 min in 5 mg/ml dispase (StemCell technologies) plus 0.1 mg/ml DNase I (Qiagen, the Netherlands) followed by filtration through a 100-mm mesh. Prior to injection in female FVB recipient mice, tumor cells were mixed with 5 μL of Matrigel (BD) and 2 μL of trypan blue solution (Sigma) and maintained on ice until injection. Surgical procedure was performed in aseptic conditions under a laminar flow hood. Mice were anesthetized with 2.5% 2-2-2-tribromoethanol (Avertin; Sigma), laid on their backs, and injected with 20 μL of cell suspension in Matrigel directly in the fourth mammary fad pad through the nipple with a Hamilton syringe. Tumor growth was monitored weekly using digital calliper, and tumor volume was calculated according to the formula: *L X W*^*2*^*/2* = *mm*^3^, where *W* represents the width and *L* the length of the tumor mass.

### BC metastatic models

Tumor resection (mastectomy) was done as previously described^37^. After anesthetizing with Avertin, the tumor mass was removed and the incision closed with wound clips. For histologic evaluation of the tumors, one part of the tumor tissue was fixed in 4% phosphate-buffered formalin and embedded in paraffin. Another part of the tumor was directly frozen in liquid nitrogen for further biological assays. One month after cell injection, mice were sacrificed by carbon dioxide inhalation. Right axillary lymph node and lung tissue were removed. To confirm the presence of metastases, sections were cut and stained with hematoxylin and eosin (H&E) as previously described^36^. In brief, for detection of metastases, the axillary lymph node and lungs were fixed in 4% phosphate-buffered formalin and embedded in paraffin. Five-micron sections of the entire lungs and lymph node were made, and slides were counterstained with H&E for the detection of metastases. The Scan Scope XT device and the Aperio Digital pathology system software were used for the analysis.

### *In vivo* therapy

Mice (n = 5 per study arm) were treated with vehicle (water) or different drugs *ad libitum* through their drinking water, starting the day after tumor injection. Daily fluid intake of fluids was monitored and fresh drugs were administered twice a week. Drug dosage in drinking water (Met 2 mg/mL, Phe 1.65 mg/mL, Asp 30 μg/mL, At 0.1 mg/mL) was based on literature data and was associated with no toxicity and no significant changes in mouse weight, and circulating levels of glucose, cholesterol and triglycerides. In mice treated with Phe, drinking water was supplemented with 5% sucrose to increase palatability.

### Immunofluorescence, confocal microscopy and immunohistochemistry analysis

Formalin-fixed paraffin-embedded samples were sectioned 5 μm thick on poly-L-lysine slides (Thermo Scientific, Waltham, MA, USA). After deparaffination and re-hydratation, antigen retrieval was obtained by heating the sections at 95 °C in 10 mM Sodium Citrate Buffer added with 0.05% Tween 20 (Sigma; pH adjusted to 6.06) for 30 minutes. Sections were then permeabilized with 1% Triton X-100 (Sigma) in PBS for 30 min at room temperature. A blocking step was performed for 30 minutes using PBS/10% BSA (Sigma). Overnight incubation at 4 °C was performed with primary antibodies diluted in PBS/BSA 10%: rabbit polyclonal antibody anti-mouse CD31 (ab28364, AbCAM, UK, 1:20 dilution) and mouse monoclonal antibody anti-α-SMA (1A4, Sigma; 1:3000 dilution). Sections were then incubated with secondary antibodies for 1 hour at room temperature (Alexa Fluor 555 Goat anti-mouse IgG2A, 1:200, Invitrogen; Alexa Fluor 488 Donkey anti-rabbit IgG, 1:200, Invitrogen, Waltham, MA, USA). Slides were stained with 4′, 6-diamino-2phenylindole (DAPI, Sigma) and mounted with Fluorashield (Sigma). Negative controls without primary antibodies were conducted for all reactions.

A preliminary scan of sections was done using a Leica SP5 II confocal microscope (Leica Microsystems, Germany) by means of a 20× oil objective in “tile scan acquisition mode”, such that all the section surface was imaged. The optical configuration was set-up as described below. For detailed confocal imaging, sequential z-stacks were collected by the Leica confocal microscope using a 40× oil immersion objective with a numerical aperture of 1.3. Fluorochromes were excited using a 405-nm diode laser for DAPI, an argon 488 laser for Alexa Fluor 488 and a 561 diode laser for Alexa Fluor 555. Detector slits were configured to minimize the cross-talk between channels and to maximize the fluorescent signal arising from the sample: PMT1, 407 to 485 nm (DAPI); PMT2, 492 to 556 nm (Alexa Fluor 488); PMT3, 579 to 684 nm (Alexa Fluor 555); the cross-talk is also limited by the fact that the stacks were collected in “between frame mode”: the blue and red fluorescence (spectrally well separated) were collected simultaneously while the green signal was obtained alone. Every single image of the collected z-stacks is 1024 × 1024 pixel^2^ (about 228 × 228 μm^2^), whereas the z-step between two images within a stack is 0.8 μm, resulting in a voxel size of 0.223 × 0.223 × 0.8 μm^3^. The acquisition parameters, the microscope, and all the detectors were controlled by means of LAS-AF software (Leica Microsystems). Quantification of blood vessel density and mean area of lumina was obtained with ImageJ analysis software.

### Statistical analysis

The Shapiro–Wilk test was used to assess for normality. Considering that the very large majority of data were not normally distributed, statistical comparisons were carried out using the nonparametric *U* test of Mann–Whitney. All reported *P* values were 2 sided.

## Additional Information

**How to cite this article**: Talarico, G. *et al.* Aspirin and atenolol enhance metformin activity against breast cancer by targeting both neoplastic and microenvironment cells. *Sci. Rep.* **6**, 18673; doi: 10.1038/srep18673 (2016).

## Supplementary Material

Supplementary Information
