# Supplementary material for: Aspirin and atenolol enhance metformin activity against breast cancer by targeting both neoplastic and microenvironment cells
Source: Sci Rep. 2016 Jan 5;6:18673. doi: 10.1038/srep18673 (PMC4700497; doi:10.1038/srep18673)
Supplement: Supplementary Information [file srep18673-s1.pdf]

Supplementary figures

Aspirin and atenolol add to metformin activity against breast cancer by targeting both neoplastic and microenvironment cells.

Giovanna Talarico[1*], Stefania Orecchioni[1*], Katiuscia Dallaglio[2], Francesca Reggiani[1], Patrizia Mancuso[1], Angelica Calleri[1], Giuliana Gregato[1], Valentina Labanca[1], Teresa Rossi[2], Douglas M. Noonan[3,4], Adriana Albini*[2], Francesco Bertolini*[1].

[1]Laboratory of Hematology-Oncology, European Institute of Oncology, Milan, Italy.

[2]Research and Statistics Department, IRCCS "Tecnologie Avanzate e Modelli Assistenziali in Oncologia" Arcispedale S. Maria Nuova, Reggio Emilia, Italy; [3]cientific and Technologic Park, IRCCS MultiMedica, Italy; [4]Department of Biotechnology and Life Sciences, University of Insubria, Varese, Italy;

[*]*These authors contributed equally*

Supplementary Fig. 1

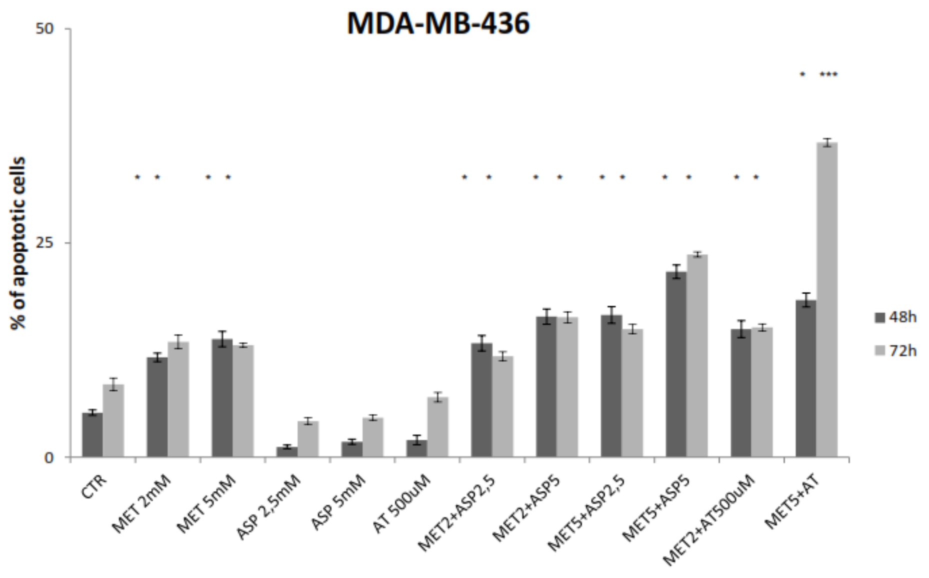

A

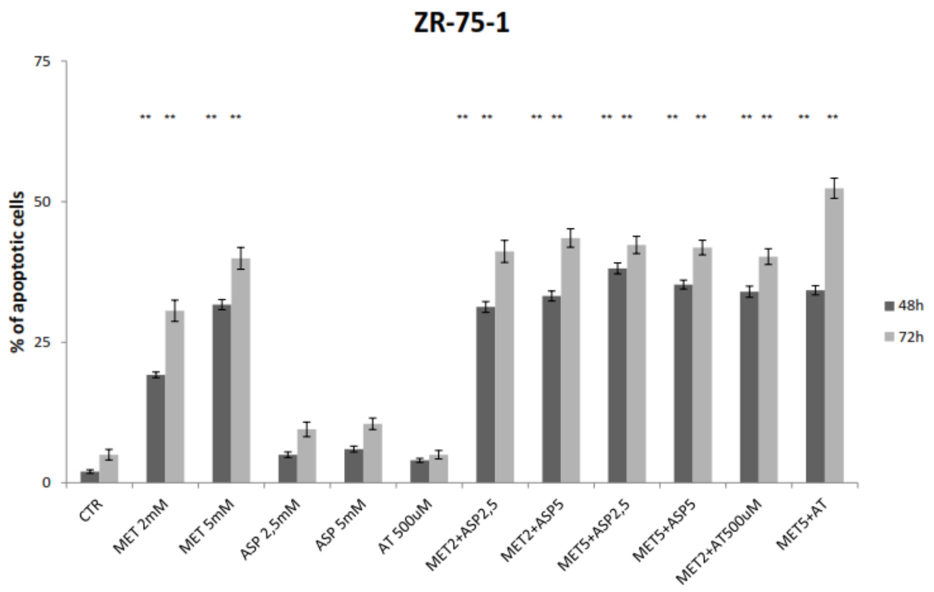

B

Supplementary Fig. 2

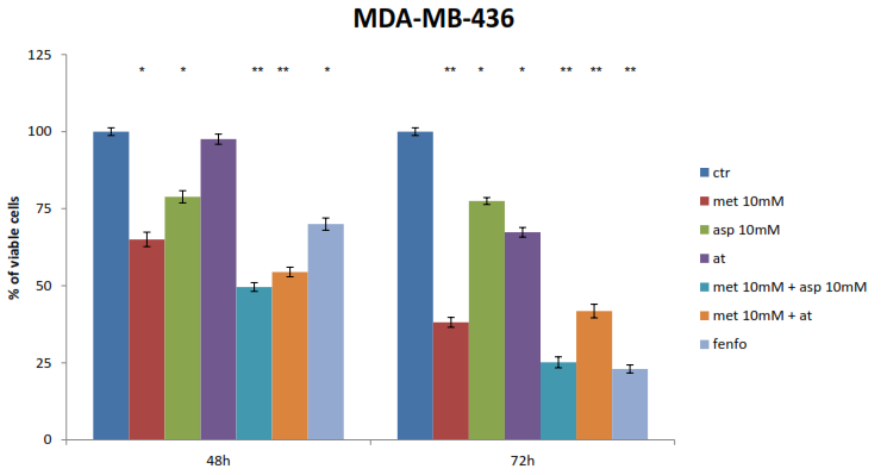

A

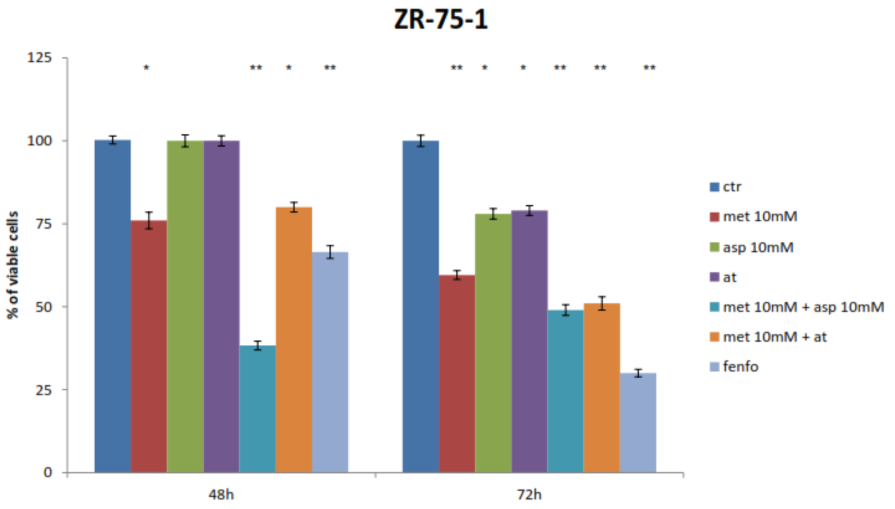

B

Supplementary Fig. 3

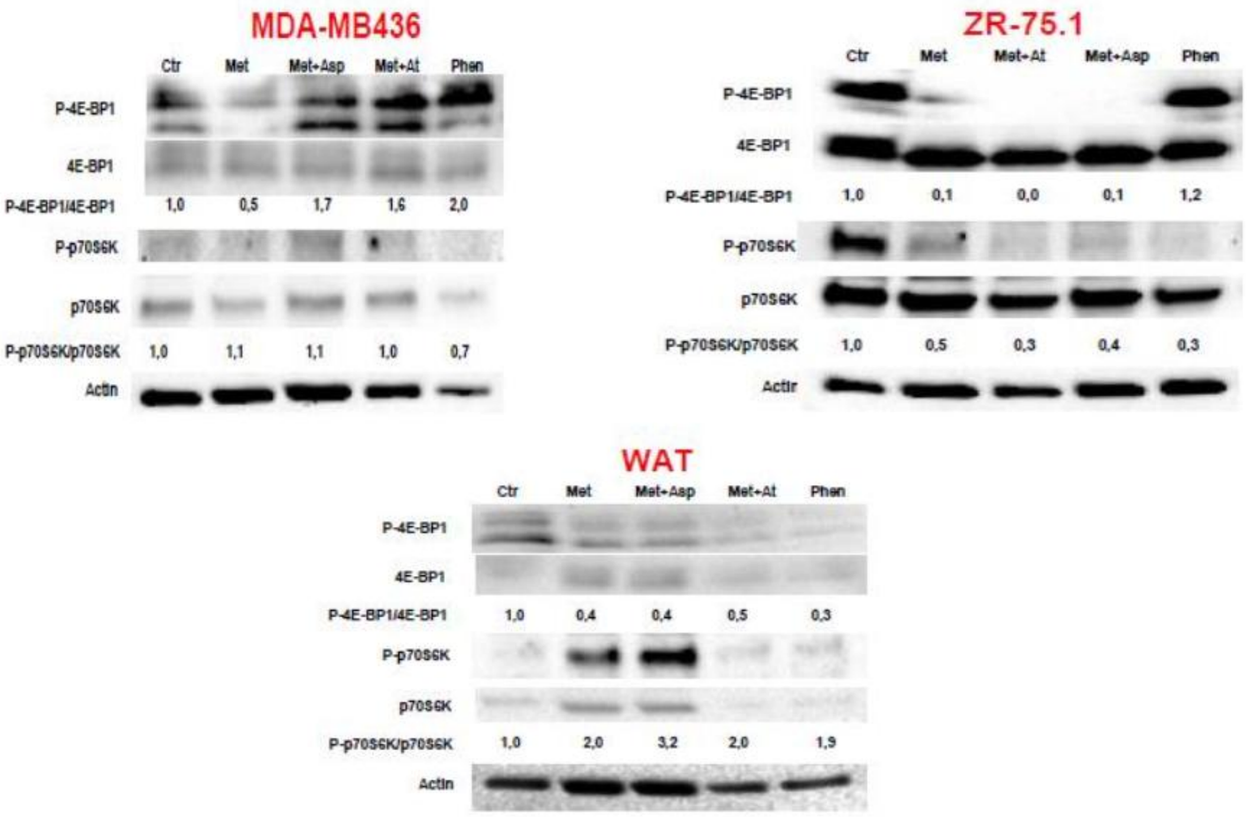

Supplementary Fig. 4

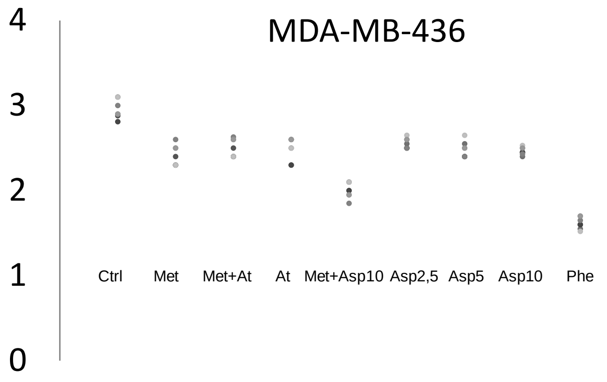

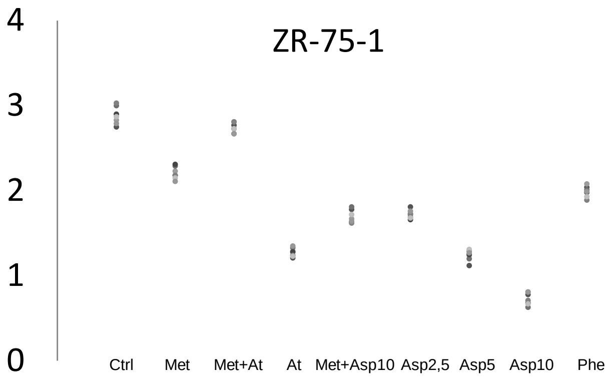

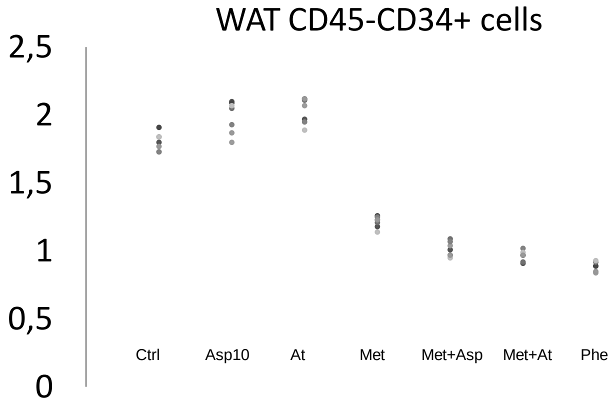

Supplementary Fig. 5

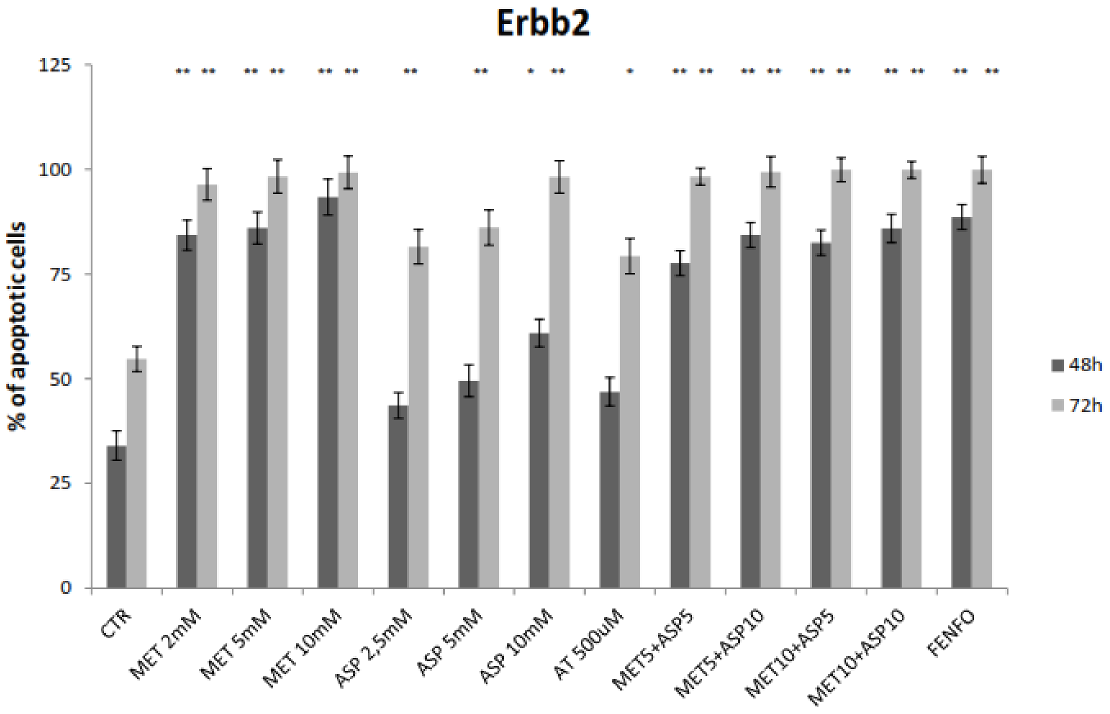

Legends of Supplementary Figures

Supplementary Fig. 1

Panel A and B show the effect of Met and Asp, alone and in combination, using lower drugs concentrations on MDA-MB-436 and ZR-75-1, respectively. Alone, Asp did not significantly increase the frequency of apoptotic cells when compared to control. An inhibitory effect of Met was observed only in ZR-75-1 cell line when used alone and in combination with Asp. Cell apoptosis was analyzed by flow cytometry.

* = P<0.05, ** = P<0.01, *** = P>0.005

Supplementary Fig. 2

Panel A and B show the effect of the different drugs, alone and in combination, on MDA-MB-436 and ZR-75-1, BC cell lines, respectively. 150.000 viable BC cells per well were seeded and treated with different combinations of Met, Phe, Asp or At. Cells were collected and counted performing a viable cell count with trypan blue.

* = P<0.05, ** = P<0.01

Supplementary Fig. 3

AMPK activation by Metformin or Met+Asp treatment is followed by the inhibition of mTOR pathway in BC cells only. 4E-BP1 and p70S6K phosphorylation by Met, Met+Asp, Met+At or Phen at the same timepoints as in Figure 2, 3 and 4. For each blot, the levels of p- 4E-BP1 over total 4E-BP1 and p- p70S6K over total p70S6K were quantified by ImageJ, compared with control (Ctr) and are represented by numbers below the bands. Actin was used as loading control.

Supplementary Fig. 4

As NAD+/NADH is a known target of biguanide activity, NAD(H) was analyzed in CD34+ WAT-derived cell and in MDA-MB-436 and ZR-75-1 BC cells using the Fluorescent NAD/NADH Detection Kit (n=7). Data are presented here as raw numbers. Asp and At alone did not show any inhibition of complex I of the respiratory chain in MDA-MB-436 and CD45-CD34+ WAT-derived progenitor cells. On the other hand, in ZR-75-1 cell lines Asp inhibits complex I in a dose dependent manner, and similar effect is obtained using At alone.

Supplementary Fig. 5

Frequency of apoptotic Erbb2 HER2+ BC cells (investigated by flow cytometry) compared to control. Cells were treated with different drug combinations including 2, 5 and 10 mM Met; 2.5, 5 and 10 mM Asp; 500uM At and 2mM Phe for 48 and 72h.

* = P<0.05, ** = P<0.01
